# Time-varying and asymmetric effects of the oil-specific demand shock on investor sentiment

**DOI:** 10.1371/journal.pone.0200734

**Published:** 2018-08-08

**Authors:** Zhifang He, Fangzhao Zhou

**Affiliations:** School of Business, Jiangnan University, Wuxi, Jiangsu, China; Central South University, CHINA

## Abstract

The relationship between oil price and investor sentiment is crucial to economic activity. Disentangling the shocks in crude oil price by structural VAR model, this paper analyzes the interaction between oil price shocks and investor sentiment by linear and nonlinear causality approach, TVP-VAR mode and NARDL model. The results reveal that changes of oil-specific demand shock not only linearly but also nonlinearly cause changes of investor sentiment while there is no significant link between other oil shocks (oil supply shock and aggregate demand shock) and investor sentiment. In addition, the study discovers that the oil-specific demand shock generally positively affects investor sentiment over time, and it has positive and asymmetric effects on investor sentiment in the short-run. In other words, it is the negative oil-specific demand shock rather than the positive component that has the significant impact on investor sentiment for short-run. This study could enrich current theories on the interaction between oil price and investor sentiment and serve as a supplement to current literature.

## Introduction

Crude oil is a core source of energy for the global economy and essential for economic activity. The implications and causes of the fluctuations in oil prices naturally draw considerable attention from a wide range of analysts and academics. Some treat oil price as a dependent variable to explore its impact on gross domestic product, consumer price, inflation, stock markets and other macroeconomic variables [1–4], while others are devoted to examining the complicated factors, such as economic growth, inventories, interest rates, US dollar exchange rate and other independent variables [5–11] that drastically affect the crude oil price. Recently, with the development of investments [12–14], investor sentiment has become a newly emerging concept, which is greatly different with these traditional fundamental factors, attracts significant attention in the crude oil market. And an increasing number of scholars have been studying the relationship between crude oil price and investor sentiment. Mainly, these studies are performed generally from two perspectives. Firstly, crude oil is one of the most important commodities in the global market, and its fluctuation is associated with investor sentiment due to the “financialization” of commodity markets. Oil has turned into an asset that equally competes with other assets for market participants’ portfolios [15]. Hence, investor sentiment in financial markets could exert certain effects on oil speculative demand and therefore oil prices. Du et al. [16] empirically document that investor sentiment helps explain the fluctuations in oil prices, as well as gasoline, heating oil and oil-company stock prices. Qadan and Nama [17] suggest that investor sentiment, captured by nine different proxies, has a significant effect on oil prices using parametric and nonparametric methods. Du and Zhao [18] provide similar international evidence that there are statistically and economically stronger effects of investor sentiment on oil prices in 2003–2008 period by a quasi-experiment setting. Deeney et al. [19] propose a measure of sentiment for West Texas Intermediate and Brent crude oil futures based on a suite of financial proxies, and show that sentiment does affect futures prices for crude oil. Maslyuk-Escobedo et al. [20] discover that price discontinuities in energy commodities related to large swings in market sentiment. The second perspective for the study about the linkage between oil price and investor sentiment is that, the fluctuation of oil price is an important driver of sentiment for investors based on the hypothesis that oil price shocks affect the general level of price, disposable incomes, expectations about current and future economic conditions, thus resulting cause investor sentiment vary. For example, Apergis et al. [21] find that energy prices, including crude oil and natural gas prices, exert a statistically significant impact on the market-based investor sentiment. Ding et al. [22] build a Chinese stock market investor sentiment index by using principal component analysis, and show that international crude oil price fluctuations significantly Granger cause Chinese stock market investor sentiment while investor sentiment could not affect crude oil price. Choi [23] provides similar evidence that investor sentiment is not useful in predicting petroleum futures returns and price changes while oil price changes play a role in predicting investor’s sentiment. Although the study about the price of oil and investor sentiment has attracted much attention, the research about the relationship between different oil price shocks and investor sentiment is extremely scarce. Since Kilian [24] points out that not all oil price shocks are alike, and the three different types of oil shocks named as oil supply shocks, aggregate demand shocks and oil-specific demand shocks are identified to capture different causes of oil price fluctuations. Therefore, different shocks in the crude oil price generate distinctly different effects on the economy and investor sentiment. Against this backdrop, we maintain that it could be desirable to extend the previous studies to assess what exactly is the linkage between investor sentiment and different oil price shocks instead of oil prices. We hold that the study is crucial to economic activity because the oil shocks and investor sentiment are key variables for policy makers, producers, industrial consumers, derivative valuation and hedging decisions.

Specifically, we take a fresh look at the relationship between oil price shocks and investor sentiment. Firstly, the price of crude oil is typically treated as exogenous with respect to the economy in existing literature. Meanwhile, it is widely accepted in recent years that the price of crude oil responds to some of the economic forces that drive investor sentiment. Therefore, it is necessary to consider the reverse causality, and consequently to redefine the relationship between oil price shocks and investor sentiment on oil price changes. Secondly, due to fact that the oil shocks and investor sentiment are both time varying and influenced by various factors, studies that do not allow the coefficients to vary over time fall short in extracting the time-varying properties of variables. Thirdly, asymmetries are widely observed in oil shocks and investor sentiment, and the findings about symmetric effects between oil price and investor sentiment will be biased towards no significant statistical relationships due to the positive and negative components of the two variables will be offset. Therefore, it is important to take account of the potentially time-varying interaction and asymmetric effect between the oil shocks and investor sentiment.

This study aims to address these existing limitations and realistic views by relating investor sentiment to measure of demand and supply shocks in the global crude oil market. A structural decomposition of the shocks in the real price of oil is built for the first step. Then, the linear and nonlinear causalities between the oil price shocks (supply shock, aggregate demand shocks and oil-specific shocks) and investor sentiment are investigated. Based on the results of causalities, their time-varying and asymmetric effects are further examined.

This paper innovates and contributes in filling some existing gaps in the literature in the following directions. First, it adds to the limited empirical findings on the interaction between oil price shocks and investor sentiment. In contrast to previous studies which only focus on the linkage between the price of oil and investor sentiment, we decomposes oil price changes into oil supply shock, global demand shock and oil specific demand based on the structural VAR model, and then the relationships between three different oil shocks and investor sentiment are examined. Second, we investigate for the first time in literature on the causalities, time-varying influence and asymmetric effects between the oil price shocks and investor sentiment. Put it simply, this paper analyses the linear and nonlinear causalities between different oil price shocks and investor sentiment by linear and nonlinear Granger causality approach, and based on the results of causalities, the time-varying influence and asymmetric effects between oil price shocks and investor sentiment are further investigated by TVP-VAR model and NARDL model, respectively. The analysis provides a rich picture on the relationship between oil price shock and investor sentiment in various dimensions (causality relationship, time-varying influence and asymmetric effect). In light of the above discussion, this paper seeks to deal with several questions emerge. Specifically, what is the causal relationship between oil price shock and investor sentiment (linear or nonlinear, bilateral or unilateral)? Further, if there is causality between the oil shock and investor sentiment, do the response of one variable to the other variable perform time-varying? Finally, do positive shocks of one variable, impact the other variable differently compared to the negative ones?

The rest of paper is organized as follows. Section 2 formally presents our econometric methodology and Section 3 discusses the data. Section 4 reports the empirical results and Section 5 offers the conclusions.

## Materials and methods

### The structural VAR model

The structural VAR model is employed to distinguish the three structural shocks that affect the real price of crude oil [24]. These are shocks to the world crude oil production (“oil supply shocks”), shocks to the global demand for all industrial commodities given by global real economic activity (“aggregate demand shocks”), and shocks to the oil demand unrelated to the global business cycle, which refers to the precautionary demand for crude oil (oil-specific demand shocks). Denote the percentage change in global crude oil production by Δ*p*_*t*_, the measure of global real economic activity by *rea*_*t*_, and the real price of oil expressed in logs by *lrpo*_*t*_. Given that some responses may be delayed by more than a year, the VAR model allows for two years’ worth of lags. Based on monthly data for *z*_*t*_ = (Δ*prod*_*t*_,*rea*_*t*_,*lrpo*_*t*_)', the structural VAR representation is as follows:

$${Α}_{0}z_{t}=\alpha +\sum_{i=1}^{24}{Α}_{i}z_{t-1}+\varepsilon_{t}$$ (1)

where *ε*_*t*_ denotes the vector of serially and mutually uncorrelated structural innovations. Suppose that the 3×1-vector of reduced-form error terms $e_{t}=A_{0}^{-1}\varepsilon_{t}$ has the following recursive representation:

$$e_{t}\equiv \left(\begin{array}{c} e_{t}{}^{\Delta prd}\\ e_{t}{}^{rea}\\ e_{t}{}^{lrpo} \end{array}\right)=\left[\begin{array}{ccc} a_{11} & 0 & 0\\ a_{21} & a_{22} & 0\\ a_{31} & a_{32} & a_{33} \end{array}\right]\left(\begin{array}{c} \varepsilon_{t}^{oil\;spply\;shock}\\ \varepsilon_{t}^{aggregate\;demand\;shock}\\ \varepsilon_{t}^{oil–specific\;demand\;shock} \end{array}\right)$$ (2)

where *ε*_*t*_^*oil spply shock*^, *ε*_*t*_^*aggregate demand shock*^ and *ε*_*t*_^*oil*–*specific demand shock*^ denote oil supply shock, aggregate demand shock and oil-specific demand shock, respectively [24]. The identification in Eq (2) implies that the aggregate demand shocks or oil-specific demand shocks do not affect oil supply contemporaneously. This reflects the view that oil producers are reluctant to change their output immediately according to changes in demand due to uncertainties in the underlying source (permanent or transitory) of the demand and the high costs of production alteration in the short run. And the aggregate demand shock reacts contemporaneously to oil supply but no change with an oil-specific demand shocks. In contrast, the real price of oil is free to respond to disruptions of oil supply and shifts in aggregate demand contemporaneously. These assumptions are consistent with classic textbook discussions. The Structural VAR Model is widely accepted to illustrate the effect of oil price shocks in a number of related contexts including stock returns [25, 26] and industrial production [27].

### Linear Granger causality test

The Linear Granger causality test by Granger [28] involves estimating a linear reduced-form vector auto regression (VAR). Let *y*_*t*_ and *x*_*t*_ (*t* = 1,2,…,*n*) be two time series, then the models could be considered as follows:

$$\begin{array}{l} y_{t}=c_{1}+\sum_{i=1}^{p}\alpha_{11i}y_{t-i}+\sum_{j=1}^{q}\alpha_{12j}x_{t-j}+u_{1t}\\ x_{t}=c_{2}+\sum_{i=1}^{p}\alpha_{21i}x_{t-i}+\sum_{j=1}^{q}\alpha_{22j}y_{t-j}+u_{2t} \end{array}$$ (3)

Where, the p and q stand for the largest lag order. The regression errors *u*_1*t*_ and *u*_2*t*_ are assumed to be mutually independent and individually i.i.d. The Granger causality method of testing for the null that *y*_*t*_ does not cause *x*_*t*_ is equivalent to testing *α*_12*j*_ = 0, *j* = 1, 2,…*q*, and the null that *x*_*t*_ does not cause *y*_*t*_ is equivalent to testing *α*_22*j*_ = 0, *j* = 1, 2,…*q*.

### Nonlinear Granger causality test

The HJ test by Hiemstra and Jones [29] is regarded as one of the best tests for nonlinear Granger causality and has been extensively applied in many fields [30–32]. However, Diks and Panchenko [33] demonstrate that the HJ test has the risk of over rejection of the null hypothesis of non-causality and propose the widely accepted DP test [34–35]. Consequently, in order to get a robust result, both the HJ and DP tests have been employed to explore the nonlinear Granger causality [36–38].

#### HJ test

To define nonlinear Granger causality, *u*_1*t*_ and *u*_2*t*_ from Eq (3) are denoted as *X*_*t*_ and *Y*_*t*_ respectively. The *m*-length lead vector matrix for *X*_*t*_ is designated by $X_{t}^{m}=(X_{t},X_{t-1},\ldots ,X_{t+m-1})$, and the *L*_*x*_-length and *L*_*y*_-length lag are denoted as $X_{t-L_{X}}^{L_{X}}=\left(X_{t-L_{X}},X_{t-L_{X}+1},\ldots ,X_{t-1}\right)$ and $Y_{t-L_{Y}}^{L_{Y}}=\left(Y_{t-L_{Y}},Y_{t-L_{Y}+1},\ldots ,Y_{t-1}\right)$, respectively. For given *m*, *L*_*x*_ and *L*_*y*_ ≥ 1, and for all *e*>0, *Y*_*t*_ does not nonlinearly Granger cause *X*_*t*_, if

$$\begin{array}{l} P\left(\left\|X_{t}^{m}-X_{s}^{m}\right\|<e|\left\|X_{t-L_{X}}^{L_{X}}-X_{s-L_{X}}^{L_{X}}\right\|<e,\left\|Y_{t-L_{Y}}^{L_{Y}}\right\|<e\right)\\ =P\left(\left\|X_{t}^{m}-X_{s}^{m}\right\|<e|\left\|X_{t-L_{X}}^{L_{X}}-X_{s-L_{X}}^{L_{X}}\right\|<e\right) \end{array}$$ (4)

Where *P*(⋅) is probability, ‖⋅‖ denotes the maximum norm, and *e* is the length scale. The test *t*-statistics has a normal distribution:

$$t=\left(\frac{C_{1}\left(m+L_{X},L_{Y},e,n\right)}{C_{2}\left(L_{X},L_{Y},e,n\right)}-\frac{C_{3}\left(m+L_{X},e,n\right)}{C_{4}\left(L_{X},e,n\right)}\right)∼N\left(0,\frac{1}{\sqrt{n}}\sigma^{2}\left(m,L_{X},L_{Y},n\right)\right)$$ (5)

where, *C*_1_, *C*_2_, *C*_3_ and *C*_4_ refer to the correlation integral estimators of the joint probabilities:

$$\begin{array}{l} C_{1}\left(m+L_{X},L_{Y},e,n\right)=\left(2/n\left(n-1\right)\right)\sum \sum_{t<s}I\left(X_{t-L_{X}}^{m+L_{X}},X_{s-L_{X}}^{m+L_{X}},e\right)I\left(Y_{t-L_{Y}}^{L_{Y}},Y_{s-L_{Y}}^{L_{Y}},e\right)\\ C_{2}\left(L_{X},L_{Y},e,n\right)=\left(2/n\left(n-1\right)\right)\sum \sum_{t<s}I\left(X_{t-L_{X}}^{L_{X}},X_{s-L_{X}}^{L_{X}},e\right)I\left(Y_{t-L_{Y}}^{L_{Y}},Y_{s-L_{Y}}^{L_{Y}},e\right)\\ C_{3}\left(m+L_{X},e,n\right)=\left(2/n\left(n-1\right)\right)\sum \sum_{t<s}I\left(X_{t-L_{X}}^{m+L_{X}},X_{s-L_{X}}^{m+L_{X}},e\right)\\ C_{4}\left(L_{X},e,n\right)=\left(2/n\left(n-1\right)\right)\sum \sum_{t<s}I\left(X_{t-L_{X}}^{L_{X}},X_{s-L_{X}}^{L_{X}},e\right) \end{array}$$ (6)

where *n* = *T*+1−*m*−max(*L*_*X*_,*L*_*Y*_), *I*(*X*,*Y*,*e*) is a basic kernel function, and its value is 1 if the modulus of the difference between *X*_*t*_ and *Y*_*t*_ are less than the bandwidth parameter *e*. Otherwise, the value is zero. And *σ*^2^(⋅) is the asymptotic variance of the test statistic.

#### DP test

Suppose that $X_{t}^{L_{X}}$ and $Y_{t}^{L_{Y}}$ are the delay vectors, there is a statement about the invariant distribution of the *L*_*X*_+*L*_*Y*_ = 1 dimensional vector $W_{t}=\left(X_{t}^{L_{X}},Y_{t}^{L_{Y}},Z_{t}\right)$, where *Z*_*t*_ = *Y*_*t*+1_. Given *W* = (*X*,*Y*,*Z*) and *L*_*X*_ = *L*_*Y*_ = 1, the joint probability density function *f*_*X*,*Y*,*Z*_(*x*,*y*,*z*) and its marginal satisfy the following relationship:

$$\frac{f_{X,Y,Z}\left(x,y,z\right)}{f_{X,Y(x,y)}}=\frac{f_{Y,Z}\left(y,z\right)}{f_{Y}\left(y\right)}$$ (7)

The restated null hypothesis implies:

$$q\equiv E\left[f_{X,Y,Z}\left(X,Y,Z\right)f_{Y}\left(Y\right)-f_{X,Y}\left(X,Y\right)f_{Y,Z}\left(Y,Z\right)\right]=0$$ (8)

where, ${\hat{f}}_{w}\left(W_{i}\right)$ is a local density estimator defined by ${\hat{f}}_{W}\left(W_{i}\right)={\left(2\varepsilon_{n}\right)}^{-d_{W}}{\left(n-1\right)}^{-1}\sum_{j,j\neq 1}I_{ij}^{W}$ with $I_{ij}^{W}=I\left(\left\|W_{i}-W_{j}\right\|<e\right)$. *I*(⋅) and *ε*_*n*_ is the indicator function the bandwidth that depends on the sample size *n*. The test statistic that is a scaled sample version of q in Eq (8) is simplified as:

$$T_{n}\left(e\right)=\frac{n-1}{n\left(n-2\right)}\sum_{i}\left({\hat{f}}_{X,Y,Z}\left(X_{i},Y_{i},Z_{i}\right){\hat{f}}_{Y}\left(Y_{i}\right)-{\hat{f}}_{X,Y}\left(X_{i},Y_{i}\right){\hat{f}}_{Y,Z}\left(Y_{i},Z_{i}\right)\right)$$ (9)

And the test statistic in Eq (9) converges to a normal distribution:

$$\sqrt{n}\frac{\left(T_{n}\left(e_{n}\right)-q\right)}{S_{n}}\to N\left(0,1\right)$$ (10)

where *S*_*n*_ is an estimator of the asymptotic variance of *T*_*n*_(⋅).

### TVP-VAR model

The TVP-VAR model of Primiceri [39] has been increasingly popular for its flexibility and robustness in capturing the time-varying properties [40–42]. It evolves from the basic structural VAR model defined as follows:

$$Ay_{t}=F_{1}y_{t-1}+\ldots +F_{s}y_{t-s}+u_{t},t=s+1,\ldots ,n$$ (11)

where *y*_*t*_ is a *k*×1 vector of observed variables, *A*,*F*_1_,…,*F*_*S*_ denote *k*×*k* matrices of coefficients, and *u*_*t*_ is a *k*×1 structural shock assumed to follow a normal distribution *u*_*t*_ ~ *N* (0,∑∑), where

$$\sum =(\begin{array}{cccc} \sigma_{1} & 0 & \ldots & 0\\ 0 & \ldots & \ldots & \ldots \\ \ldots & \ldots & \ldots & 0\\ 0 & \ldots & 0 & \sigma_{k} \end{array})$$ (12)

To specify the simultaneous relations of the structural shock by recursive identification, *A* as a lower-triangular is defined as follows:

$$A=(\begin{array}{cccc} 1 & 0 & \ldots & 0\\ a_{21} & \ldots & \ldots & \ldots \\ \ldots & \ldots & \ldots & 0\\ a_{k1} & \ldots & a_{k,k-1} & 1 \end{array})$$ (13)

The Eq (11) can be written as the following reduced form VAR model:

$$y_{t}=B_{1}y_{t-1}+\ldots +B_{S}y_{t-s}+A^{-1}\sum \varepsilon_{t},\;\varepsilon_{t}∼N\left(0,I_{K}\right)$$ (14)

where *B*_*i*_ = *A*^−1^*F*_*i*_,*i* = 1,…,*s*. *β* is a (*k*^2^*s*×1) vector obtained by stacking the elements in the rows of *B*_*i*_'*s*, and letting $X_{t}=I_{k}⊗\left(y_{t-1}^{\prime },\ldots ,y_{t-s}^{\prime }\right)$, where ⊗ denotes the Kronecker product, then Eq (14) can be rewritten as follows:

$$y_{t}=X_{t}\beta +A^{-1}\sum \varepsilon_{t}$$ (15)

Now, all parameters in Eq (15) are time varying. We further extend it to the following specification by allowing the parameters to change over time:

$$y_{t}=X_{t}\beta_{t}+A_{t}^{-1}\sum_{t}\varepsilon_{t}t=s+1,\ldots ,n,$$ (16)

where *β*_*t*_, *A*_*t*_ and ∑_*t*_ are all time varying. Let *a*_*t*_ = (*a*_21_,*a*_31_,*a*_32_,*a*_41_,…*a*_*k*,*k*−1_)′ denote a stacked vector of the low-triangular elements in *A*_*t*_ and *h*_*t*_ =(*h*_1*t*_,…,*h*_*kt*_)′, $h_{jt}=\log\sigma_{jt}^{2}$, *j* = 1,…,*k*, *t* = *s*+1,…,*n*. It is assumed that the parameters in Eq (16) follow a random walk process:

$$\begin{array}{c} \beta_{t+1}=\beta_{t}+u_{\beta t}\\ a_{t+1}=a_{t}+u_{at}\\ h_{t+1}=h_{t}+u_{ht} \end{array}\left(\begin{array}{l} \varepsilon_{t}\\ u_{\beta t}\\ u_{at}\\ \mu_{ht} \end{array}\right)∼N\left(0,\left(\begin{array}{cccc} I & O & O & O\\ O & \sum {}_{\beta } & O & O\\ O & O & \sum {}_{a} & O\\ O & O & O & \sum {}_{h} \end{array}\right)\right)$$ (17)

where *t* = *s*+1,…,*n*, *β*_*s*+1_ ~ *N* (*u*_*β*0_,∑_*β*0_), *a*_*s*+1_ ~ *N* (*u*_*a*0_,∑_*a*0_), and *h*_*s*+1_ ~ *N* (*u*_*h*0_,∑_*h*0_).

### NARDL model

The NARDL model proposed by Shin et al. [43] has been extensively used in many fields [32, 44, 45]. It is common that dynamic relationships between first-order integrated variables can be reproduced via an Error Correction Model (ECM) [46–47] that takes the following form:

$$\Delta y_{t}=\mu +\rho_{y}y_{t-1}+\rho_{x}x_{x-1}+\sum_{i=1}^{p-1}\alpha_{i}\Delta y_{t-i}+\sum_{i=0}^{q-1}\beta_{i}\Delta x_{t-i}+\varepsilon_{t}$$ (18)

Where *y*_*t*_ and *x*_*t*_ denote the dependent and independent variables, respectively. The symbol Δ denotes first differences. Even though the Eq (18) enables the investigation of the short- and long-run links between the variables, it becomes impertinent and will be misspecified when these relationships are nonlinear and/or asymmetric. In this context, the term “hidden cointegration” is introduced, which is detected if two-time series have hidden cointegration and if their positive and negative components are cointegrated with each other [48]. That is why a nonlinear and asymmetric ECM is of great interest. The cointegrating NARDL model uses positive and negative partial sum decompositions to allow for the detection of asymmetric effects both in the long- and the short-run. The positive and negative partial sums, i.e., $x_{t}^{+}$ and $x_{t}^{-}$, of increases and decreases such as

$$x_{t}^{+}=\overset{t}{\underset{j=1}{\sum }}\Delta x_{j}^{+}=\overset{t}{\underset{j=1}{\sum }}\max(\Delta x_{j},0);x_{t}^{‑}=+\overset{t}{\underset{j=1}{\sum }}\Delta x_{j}^{‑}=\overset{t}{\underset{j=1}{\sum }}\min(\Delta x_{j},0)$$ (19)

The Eq (18) is expanded to the general NARDL model expressed as follows:

$$\Delta y_{t}=\mu +\rho_{y}y_{t-1}+\rho_{x}^{+}x_{t-1}^{+}+\rho_{x}^{-}x_{t-1}^{-}+\sum_{i=1}^{p-1}\alpha_{i}\Delta y_{t-i}+\sum_{i=0}^{q-1}(\beta_{i}^{+}\Delta x_{t-i}^{+}+\beta_{i}^{-}\Delta x_{t-i}^{-})+\varepsilon_{t}$$ (20)

The superscripts (+) and (–) in Eq (20) stand for the positive and negative partial sums decomposition as defined above. The p and q stand for the lag order. The positive and negative impact of exogenous variable on dependent variable can be reflected by $\rho_{x}^{+}$ and $\rho_{x}^{-}$, respectively, and the long-run symmetry can be tested by the null hypothesis $\rho_{x}^{+}=\rho_{x}^{-}$. The positive and negative long-run coefficients can be computed as $\theta^{+}=‑\rho_{x}^{+}/\rho_{y}$ and $\theta^{+}=‑\rho_{x}^{-}/\rho_{y}$. The short-run adjustment to a positive and a negative shock of exogenous variable is captured by $\beta_{i}^{+}$ and $\beta_{i}^{-}$, respectively. The short-run symmetry can be equally tested by the null hypothesis $\beta_{i}^{+}=\beta_{i}^{-}$ for all *i* = 0,….,*q*−1. The non-rejection of either the long-run symmetry or the short-run symmetry will yield the cointegrating NARDL model with short-run asymmetry in Eq (21) and with long-run asymmetry in Eq (22), respectively:

$$\Delta y_{t}=\mu +\rho_{y}y_{t-1}+\rho_{x}x_{t-1}+\sum_{i=1}^{p-1}\alpha_{i}\Delta y_{t-i}+\sum_{i=0}^{q-1}(\beta_{i}^{+}\Delta x_{t-i}^{+}+\beta_{i}^{-}\Delta x_{t-i}^{-})+\varepsilon_{t}$$ (21)

$$\Delta y_{t}=\mu +\rho_{y}y_{t-1}+\rho_{x}^{+}x_{t-1}^{+}+\rho_{x}^{-}x_{t-1}^{-}+\sum_{i=1}^{p-1}\alpha_{i}\Delta y_{t-i}+\sum_{i=0}^{q-1}\beta_{i}\Delta x_{t-1}+\varepsilon_{t}$$ (22)

## Data analysis

The oil market variables (world crude oil production, global real economic activity, the real price of crude oil) and investor sentiment index are used for our analysis. The world crude oil production and the price of crude oil are obtained from the U.S. Energy Information Administration (EIA). Specifically, the monthly data on world crude oil production in thousand barrels per day is available from the U.S. EIA Monthly Energy Review. Following the preceding literatures, the world crude oil production enters our vector of endogenous variables in terms of annualized percentage changes, denoted by Δ*prod*_*t*_. The real price of oil is the spot Western Texas Intermediate (WTI) crude oil price deflated by the U.S. CPI, expressed in logs and denoted by *lrpo*_*t*_. The global real economic activity is measured by the real economic activity index created by Kilian [24], which is based on single-voyage dry cargo ocean shipping freight rates and expressed in terms of the deviation of real freight rates from their long-run trend. The real economic activity index is obtained from Kilian’s website: http://www-personal.umich.edu/~lkilian/. Given that the real economic activity index is a global business cycle measure and stationary by construction, it enters the SVAR model in levels and denoted by *rea*_*t*_.With respect to the investor sentiment, it is a belief about future cash flows and investment risks that is not justified by economic fundamentals [49]. And being a belief about future cash flows and investment risks, it is not justified by economic fundamentals. Thus, the sentiment indexes developed by Baker and Wurgler are not derived from oil market conditions, which make these sentiment measures more likely to be exogenous to oil fundamentals [18]. It is designed specifically to capture the market-wide investor sentiment in financial markets, and is free of idiosyncratic noise in individual sentiment measures. Therefore, in this paper, the Baker and Wurgler's [49, 50] monthly sentiment index are used as the proxy of investor sentiment from Wurgler's website: http://people.stern.nyu.edu/jwurgler/. All the monthly series for this empirical study are from January 1986 to September 2015, including 357 samples. This sample period is determined by the availability of WTI crude oil price starting on January 1987, and the BW investor sentiment index is available from July1965 to September 2015.

**Fig 1:**
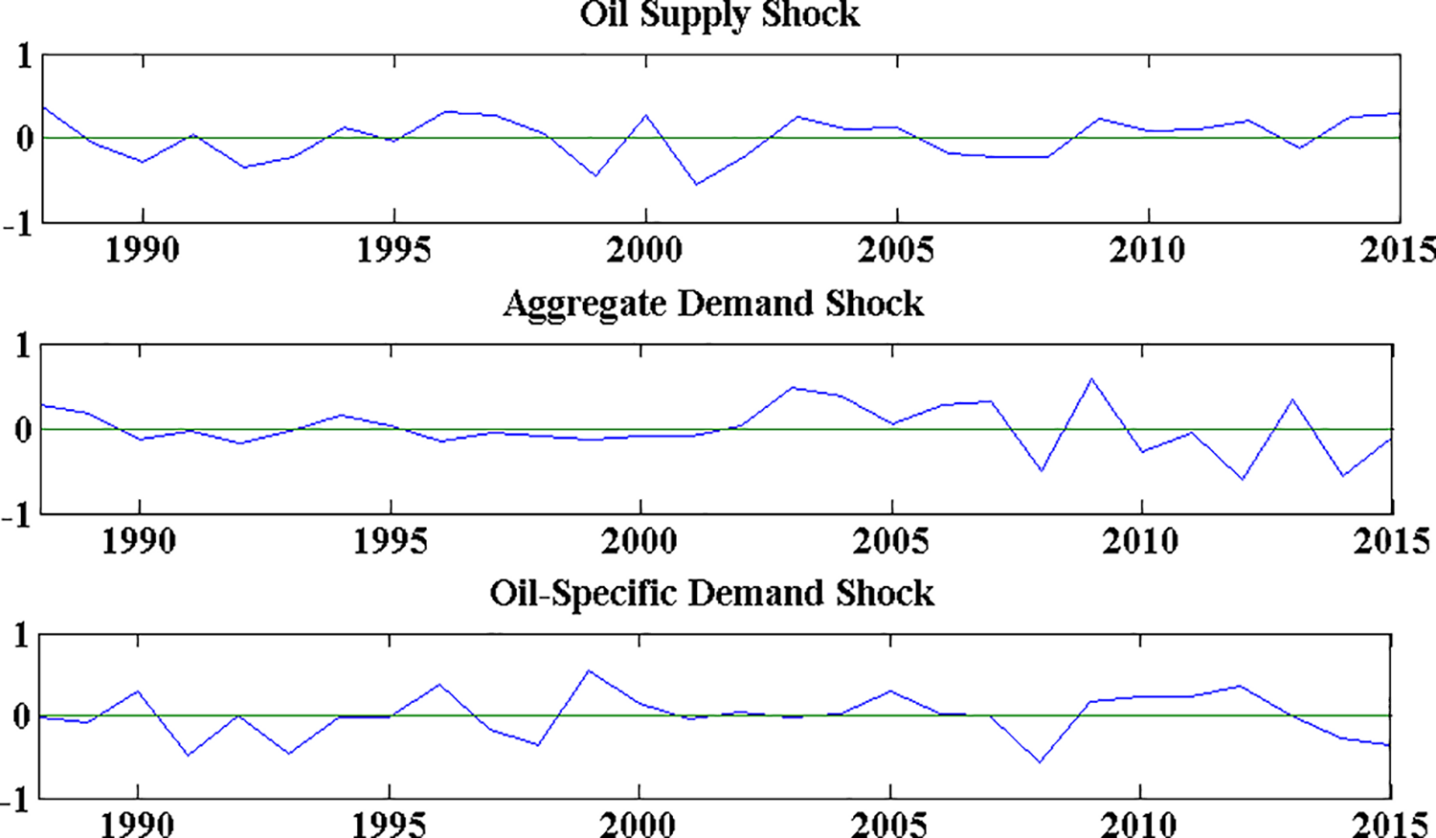
Historical evolution of structural oil supply and demand shocks.

The oil supply shock, aggregate demand chock and oil-specific shock are obtained from the estimation of the structural VAR model. Fig 1 plots annual averages for structural oil supply and demand shocks. As in Kilian’s study [24], we annualize the monthly shock series to facilitate their readability. It can be seen that the real price of crude oil is driven by a time-varying combination of oil supply, aggregate demand, and oil-specific demand shocks. For example, the top panel of Fig 1 exhibits small negative oil supply shock in 1990, this supply shock is accompanied by a small negative aggregate demand shock, whereas a large positive oil-specific demand is associated with Persian Gulf War in 1900. The oil supply shock becomes large in 1997 with the Asian Financial Crisis, followed by a small negative aggregate oil demand shock and oil-specific demand shock due to the global business cycle. However, there is an oil-specific demand shock peak in 1999, which refers to the historical low price of crude oil during that time. It is also confirmed by the low oil supply shock in 1999. The large fluctuation of oil supply shock around 2000 is influenced by the great fluctuation of oil price during that period. As is known, the price of crude oil briefly raised above $30 a barrel but quickly fell below $20 a barrel in 2000. Another obvious feature in Fig 1 is that the oil aggregate demand shock and oil-specific demand shock both reach great negative levels in 2008 which is attributed to the global financial crisis. And the negative oil supply shock in that time also obviously reflects the effect of global economic crisis. After 2012, the oil-specific shock maintain negative and the oil supply shock is positive, while the aggregate demand shock has prominent change during 2012–2013 that may be affected by the European debt crisis.

Table 1 reports the descriptive statistics and statistical properties of the oil supply shock, aggregate demand, oil-specific demand shock and investor sentiment. It can be identified that the oil supply shock, aggregate demand shock, oil-specific demand shock and investor sentiment exhibit small variability over the period, as indicated by their respective standard deviations. All the series display negative skewness with the exception of investor sentiment which is positively skewed. The Jarque-Bera (JB) test for normality shows that all the series depart from the normal distributions, except the oil-specific demand shock. The stationary of the variables is investigated by conducting Augmented Dicky-Fuller (ADF), Dicky-Fuller GLS (DFGLS), Phillips-Perron (PP), Elliott-Rothenberg-Stock Point-Optimal (ERS) and Kwiatkowski-Phillips-Schmidt-Shin (KPSS) tests. The obtained results indicate all the series are stationary at 10% significant level. Although the ADF test of investor sentiment cannot reject the null hypothesis at 10% significant level, results of other four tests (DDFGL, PP, ERS, KPSS) clearly show the stationary investor sentiment.

**Table 1:** Linear Granger causality between oil price shocks and investor sentiment.

Variables	Oil supply shock	Aggregate demand shock	Oil-specific demand shock	Investor sentiment
Mean	0.0005	0.0011	0.0008	0.2077
Median	0.0504	0.0447	0.0016	0.1100
Maximum	3.5217	3.4700	2.8742	3.0800
Minimum	-5.5706	-3.86653	-2.9274	-0.8700
St.dev	0.8833	0.8835	0.8833	0.5946
Skewness	-0.7883	-0.5419	-0.1323	1.6032
Kurtosis	7.9920	7.0992	3.1303	7.5865
JB statistic	380.2569***	249.4436***	1.2075	434.5211***
ADF test	-18.2518***	-18.1576***	-18.0637***	-2.4507
DFGLS test	-17.8520***	-16.5123***	-18.0363***	-2.4206**
PP test	-18.2518***	-18.1575***	-18.0723***	-2.9726**
ERS test	0.1508***	0.2119***	0.1966***	2.1828**
KPSS test	0.0456***	0.1178***	0.1545***	0.2641*

***, **,and * denot the significance at 1%, 5%, and 10% level, respectively.

The null hypotheses for ADF, DFGLS and PP test are that the series has a unit root I(1).

The null hypothesis of the ERS and KPSS test is that the series is stationary I(0).

## Results

### Linear Granger causality test

The results of linear Granger causality test between the oil price shocks and investor sentiment depending on VAR in Eq (3) are shown in Table 2. Lags refer to the number of months by which one variable lags another variable. The optimal lag length is 1 that is proposed by the sequential modified LR test statistic (LR), the Schwarz information criterion (SC) and the Hannan-Quinn information criterion. In order to illustrate more clearly the relationship between crude oil return and investor sentiment more clearly and examine the robustness, the lagged number is selected from 1 to 4. The results suggest that there is neither the significant causality between oil supply shock and investor sentiment, nor causal relationship between aggregate demand shock and investor sentiment. However, the non-causality from oil-specific demand shock to investor sentiment is rejected at 5% level, while the non-causality from investor sentiment to oil-specific demand shock is rejected with all lags order. The findings indicate that the oil supply and aggregate demand shock are not related to investor sentiment, but changes in the oil-specific demand that are more related to unexpected and precaution demand can cause investor sentiment fluctuated. As is known, oil supply shock and aggregate demand are mainly determined by economic fundamentals. In contrast, financial investor sentiment is exogenous to economic fundamentals. This may be the main reason for the non-causality results. With respect the unilateral linear causality from oil-specific demand shock to the investor sentiment, it can be interpreted in two ways. On the one hand, changes in oil-specific demand will release a signal that the market has been disturbed by some specific information and the original balance is upset. Hence, the investor sentiment will vary subsequently to changes in oil-specific demand. On the other hand, oil-specific demand generally induced by some specific oil-related events leads to more unstable market expectations and changes of the investor sentiment. In fact, the oil-specific demand is influenced by many factors and information from market. And investor sentiment, as a psychological factor in market, it is too weak to cause the change of on oil-specific demand.

**Table 2:** Linear Granger causality between oil price shocks and investor sentiment.

H_0_	Lags	F-statistic	Prob.	H_0_	Lags	F-statistic	Prob.
Oil supply shock ↛ Investor sentiment	1	3.2601	0.0719	Investor sentiment ↛ Oil supply shock	1	1.3678	0.2430
	2	1.7977	0.1673		2	1.2263	0.2947
	3	1.6638	0.1747		3	0.8075	0.4905
	4	1.3075	0.2670		4	0.6910	0.5987
Aggregate demand shock ↛ Investor sentiment	1	0.0547	0.8153	Investor sentiment ↛ Aggregate demand shock	1	0.5152	0.4734
	2	0.3971	0.6726		2	0.9423	0.3908
	3	0.5967	0.6176		3	0.7221	0.5394
	4	0.4359	0.7827		4	0.5610	0.6912
**Oil-specific demand shock** **↛ Investor sentiment**	1	6.9139***	**0.0090**	Investor sentiment ↛ Oil-specific demand shock	1	1.4525	0.2290
	2	3.5393**	**0.0302**		2	0.9189	0.4000
	3	2.8828**	**0.0360**		3	1.2199	0.3025
	4	2.7873**	**0.0266**		4	1.2798	0.2777

***, **,and*denote significance at 1%, 5%, and 10% level, respectively. H_0_ is the null hypothesis. ↛ denotes the null hypothesis that the left variable cannot Granger cause the right variable

However, the linear Granger causality test cannot capture nonlinear and higher-order causal relationships. To capture the potential higher-order effects, we consider a nonlinear Granger-causality test. Specifically, we use the most commonly used HP and DP tests to examine the nonlinear causality. To compute HJ statistic, the lag lengths *L*_*x*_ and *L*_*y*_, and the distance measure *e* must be selected. We set *e* = 1.5 for both series and the lag length *L*_*x*_ = *L*_*y*_ [31,32,40], and the lag lengths are selected from 1 to 4. The results of both the HJ and DP for analyzing nonlinear causality between oil price shocks and investor sentiment are presented in Table 3.

**Table 3:** Nonlinear Granger causality between oil price shocks and investor sentiment.

	Oil supply shock ↛Investor sentiment							Investor sentiment ↛Oil supply shock			
Lags	HJ	Prob.		DP			Prob.	HJ	Prob.	DP	Prob.
1	0.2295	0.4093		0.3433			0.3657	1.2107	0.1130	1.2675	0.1025
2	0.1070	0.4574		0.3166			0.3758	0.5727	0.2834	0.4801	0.3156
3	0.0384	0.4847		0.2238			0.4115	0.4711	0.3188	0.3627	0.3584
4	0.0728	0.4710		0.3911			0.3479	-0.2349	0.5928	-0.4190	0.6624
	Aggregate demand shock ↛Investor sentiment							Investor sentiment ↛Aggregate demand shock			
Lags	HJ	Prob.	DP			Prob.		HJ	Prob.	DP	Prob.
1	-0.3535	0.6382	-0.0630			0.5251		-0.5986	0.7253	-0.2036	0.5807
2	-0.5367	0.7043	-0.0457			0.5182		-1.0417	0.8513	-0.6843	0.7531
3	-0.7353	0.7689	-0.2628			0.6036		-1.2386	0.8922	-0.9481	0.8285
4	-0.7904	0.7854	-0.3061			0.6202		-0.8759	0.8095	-0.5314	0.7024
	**Oil-specific demand shock ↛Investor sentiment**							Investor sentiment ↛Oil-specific demand shock			
Lags	HJ	Prob.	DP		Prob.			HJ	Prob.	DP	Prob.
1	1.2552	**0.1047**	1.0726		0.1417			0.2979	0.3829	0.2645	0.3957
2	1.8332	0.03340**	1.9271		0.0270			0.2365	0.4065	0.1555	0.4382
3	1.5683	0.0584*	1.5642		0.0588			-0.0131	0.5052	-0.0486	0.5194
4	1.1390	**0.1274**	1.0652		0.1434			-0.1209	0.5481	0.0009	0.4997

As expected, there is still no proof that oil supply shock and aggregate demand shock can nonlinearly lead investor sentiment, and that investor sentiment can nonlinearly lead to the three oil shocks according to the results of Table 3. In addition, the bi-directional nonlinear Granger causality from oil-specific demand shock to investor sentiment is confirmed with lags of 2 and 3, and the results can support the hypothesis that oil-specific demand shock leads to investor sentiment changes for lags 1 and 4 at the significance level of 15% instead of 10%. Overall, we further find the evidence that there is only a significant linear and nonlinear causality from oil-specific demand to the investor sentiment, while no proof to support the existing linear and nonlinear causality between other oil shocks and investor sentiment.

Therefore, in the following section, we focus on the study of how the oil-specific demand shock affects investor sentiment. In fact, the effect of oil-specific demand shock on investor sentiment is complicated. Firstly, the changes between oil price and investor sentiment over time may lead the effect is time varying. For example, it is well known that the international oil prices experienced rollercoaster-ride of fluctuation during the 2008 financial crisis followed by a significant soaring as a result of Libyan war in 2011. And it has also been confirmed that investor sentiment has a lower level during financial crisis in 2008 while a higher level during a rapid economic development period. Another underlying reason for the complicated effect of oil-specific demand shock on investor sentiment may be the asymmetric influence. The negative oil-specific demand shock and the positive oil-specific demand shock may generate different effects on investor sentiment, resulting in an asymmetric effect. The oil price is usually divided into the positive and negative ones to examine the asymmetric effect on asset prices [44, 51]. These characteristics imply there may be a time-varying and asymmetric effect of oil-specific demand shock on investor sentiment. To carry out our investigation, a TVP-VAR model and NARDL model are further employed to explore the evolving roles of oil-specific demand in investor sentiment.

### Time-varying influence

We focus upon the time-varying effect of oil-specific demand shock on investor sentiment using the TVP-VAR model. The TVP regression model is estimated by Markov Chain Monte Carlo (MCMC) algorithm in the context of a Bayesian inference. We draw M = 20000 sample after initial 2000 sample are discarded for convergence, and perform diagnostic tests for convergence and efficiency. Table 4 presents the estimations for posterior means, standard deviations, the 95-persent credible intervals, the convergence diagnostics (CD) and inefficiency factors according to Nakajima [41]. It cannot reject the null hypothesis of convergence to the posterior distribution at the conventional level significance. In addition, we observe quite low inefficiency factors, and the maximum inefficiency factor is 143.71, implying about 20000/143.71 = 139 uncorrelated samples that is considered to be sufficient for the posterior inference. These characteristics confirm the efficiency of the MCMC algorithm in replicating the posterior draws.

**Table 4:** Estimation results of TVP-VAR model.

Parameter	Mean	SD	95% Confidence	Geweke’s CD	Inefficiency factor
${\left(\sum {}_{\beta }\right)}_{1}$	0.0023	0.0002	[0.0019,0.0026]	0.260	9.53
${\left(\sum {}_{\beta }\right)}_{2}$	0.0023	0.0002	[0.0019,0.0026]	0.730	9.79
(∑_*a*_)	0.0046	0.0010	[0.0031,0.0068]	0.347	53.78
${\left(\sum_{h}\right)}_{1}$	0.0067	0.0024	[0.0035,0.0130]	0.850	143.71
${\left(\sum_{h}\right)}_{2}$	0.1594	0.0316	[0.1047,0.2316]	0.262	55.47

**Fig 2:**
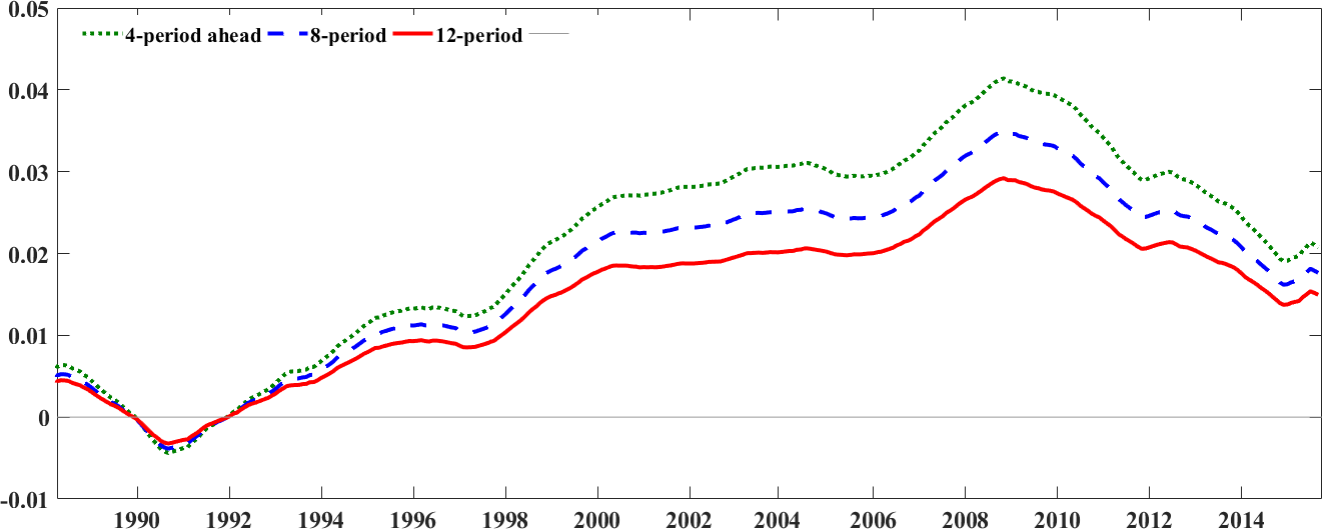
TVP-VAR impulse responses of investor sentiment under oil-specific demand.

According to the results of linear and nonlinear Granger causality, we find that only the oil-specific demand shock can affect investor sentiment, but there is no causality between other variables. Therefore, the effect oil-specific demand shock on investor sentiment is further studied. Specifically, to capture the dynamic influence of oil-specific demand shock on investor sentiment over time, we use the impulse response function based on the estimated TVP-VAR model by showing the responses for each selected horizon (i.e., 4 periods, 8 periods and 12 periods ahead) at all points in time. Fig 2 displays the time varying responses of investor sentiment to oil-specific demand shock. We find that the oil-specific demand shock exhibits a significant positive impact on investor sentiment over different horizons and over time with the exception of the period during 1990–1992, when the impulse response exerts an insignificant yet negative effect on investor sentiment. It is associated with the Gulf War, when disruption of the oil supply in Iran and Traq triggered a persistent oil price hike, which resulted in the oil-specific demand shocks such as precautionary demand shock increasing and raised uncertainties and fear about future energy market conditions. The aggregate fear finally affects investment behavior, resulting in reduced or postponed investment and low sentiment. After 1992, the impact of oil-specific demand shock on investor sentiment becomes positive again, and continues to strengthen until 2009, implying the increasing effect of oil-specific demand shock on investor sentiment. In addition, it is easy to understand the positive effect of specific oil demand shock on investor sentiment. On one hand, a growing oil demand will play an important role in the increase of the oil price, and the increasing oil price is often accompanied by high investor sentiment. On the other hand, the oil-specific demand is usually viewed as a non-fundamental and speculative demand that is triggered by the expectation of changes in crude oil market taking place in the future. Therefore, the increased oil-specific demand shock implies the increasing forward-looking demand activity has taken place, and it results in an increase in investor sentiment in crude oil market. As it can be seen that, the positive response of investor sentiment to oil-specific demand gradually decline after 2009.This may be due to the Global financial crisis that brought a profound and persistent impact not only on the oil-specific demand but also on investor sentiment. In particular, the unprecedented drop in oil prices after the Great Recession seems to be driven by the moderate oil-specific demand shock and speculation. And investors are more cautions after experiencing the Great Recession, which leads to their sentiment as a response to oil-specific demand shock becomes gradually moderate.

### Asymmetric effects

We estimate the specification models developed in Eqs (18)–(22) to further examine the symmetric and asymmetric impacts of oil-specific demand shock on investor sentiment, and perform the Wald test for detecting the short-run (*W*_*SR*_) and long-run (*W*_*LR*_) symmetry. The preferred specification is chosen with max p = max q = 12 by dropping all insignificant stationary regressors. The obtained results are reported in Table 5. Where, *OSD* and *BW* denote the oil-specific demand shock and investor sentiment, respectively. And *L*_*OSD*+_ and *L*_*OSD*−_ indicate the positive and negative long-run coefficients, respectively. Estimation for the symmetric ARDL shows that previous oil-specific demand shock has a significantly positive impact on investor sentiment, and this result is consistent with that of TVP-VAR model. The results from the NARDL with the long-run asymmetry model show that the estimated long-run coefficients for both positive and negative coefficients of oil-specific demand shock are not significant at 10% level. The Wald test for long-run symmetry *W*_*LR*_ cannot be rejected for the null hypothesis, indicating both the positive and negative oil-specific demand shock could not exert significant impact on investor sentiment in the long-run. However, according to the results of the NARDL with short-run asymmetry model, it can be seen clearly that, in the short-run, a decrease of 1% in the oil-specific demand shock entails a cumulative decrease of 0.0470% in investor sentiment while the positive component cannot be seen in the results. These evidences suggest that the negative oil-specific demand shock is a main and an important driver of investor sentiment while the negative oil-specific demand shock play an inconsequential role in investor sentiment in the short-run. What’s more, the asymmetric effect is confirmed by the Wald test statistic (*W*_*SR*_), suggesting that the change of oil-specific demand shock has asymmetric effects on investor sentiment in short-run. Estimation of the NARDL with short-run and long-run asymmetry model also displays that both the positive and negative coefficients in the long-run are insignificant. And we fail to detect the asymmetric (negative versus positive) long-term effects of oil-specific demand on investor sentiment from the Wald test for long-run (*W*_*LR*_), which further confirms that investor sentiment is not sensitive to the oil-specific demand shock in the long-run. Effectively, as it can be seen that, in the short-run, the negative component of oil-specific demand shock is significant and positive (0.0356+0.0446 = 0.0802) while no positive component is shown in the results. Furthermore, the Wald test statistic (*W*_*SR*_) clearly rejects linearity. It also suggests that oil-specific demand shock decreases are passed onto investor sentiment in the short-run and it is a more important driver of investor sentiment than the increases of oil-specific demand shock. Overall, our estimations support the idea that the oil-specific demand shock cannot exert influence on investor sentiment in the long run while it does have significant and asymmetric effects on investor sentiment in the short-run, and this significant impact is originated mainly from the negative oil-specific demand instead of the positive one. It is easily to understand that the significant effect of oil-specific shock has effects on investor sentiment only appears in the short-run. Since the oil-specific demand shocks reflect in particular fluctuations in precautionary demand for oil driven by fears about future oil supplies as pointed by Kilian [24], it can affect investor sentiment immediately and directly when there are great fluctuations for oil-specific demand shocks. However, these great fluctuations are gradually diluted and absorbed by various factors even including investor sentiment as time goes on. Therefore, the impact of oil specific demand is weakened over time and resulting in the non-significant effects on investor sentiment in the long run. Additionally, the reason for the finding that it is the negative oil-specific demand shock instead of the positive one significantly affects investor sentiment, may relate to the “leverage effect” in financial market. That is, the shock of negative information usually has stronger impact on stock price than that of negative information because investors are more sensitive to negative news than positive news. Due to the “financialization” of commodity markets, there may also be “leverage effect” in oil market, which resulting in the positive oil specific demand shock has more significant influence on investor sentiment than the negative one.

**Table 5:** Estimation for asymmetric effects of oil-specific demand shock on investor sentiment.

Symmetric ARDL		NARDL with long-run asymmetry		NARDL with short-run asymmetry		NARDL with short-run and long-run asymmetry	
*BW*_*t*−1_	-0.0623***	*BW*_*t*−1_	-0.0769***	*BW*_*t*−1_	-0.0479***	*BW*_*t*−1_	-0.0556***
*OSD*_*t*−1_	0.0224**	$OSD_{t-1}^{+}$	0.0001	*OSD*_*t*−1_	0.0258***	$OSD_{t-1}^{+}$	0.0003
Δ*BW*_*t*−4_	0.1127**	$OSD_{t-1}^{-}$	0.0005	Δ*BW*_*t*−7_	0.2148***	$OSD_{t-1}^{-}$	0.0004
Δ*BW*_*t*−7_	0.2350***	Δ*BW*_*t*−4_	0.1222**	Δ*BW*_*t*−11_	0.1477***	Δ*BW*_*t*−7_	0.2239***
Δ*BW*_*t*−11_	0.1347** (2.4097)	Δ*BW*_*t*−7_	0.2415***	Δ*BW*_*t*−12_	-0.1283**	Δ*BW*_*t*−11_	0.1481***
*μ*	0.0137	Δ*BW*_*t*−10_	0.1105**	$\Delta OSD_{t-4}^{-}$	0.0470***	Δ*BW*_*t*−12_	-0.1173**
		Δ*BW*_*t*−11_	0.1329**	*μ*	0.0272**	$\Delta OSD_{t-1}^{-}$	0.0356**
		Δ*OSD*_*t*−1_	0.0152**			$\Delta OSD_{t-4}^{-}$	0.0446***
		*μ*	0.0441*			*μ*	0.0634***
		*L*_*OSD*_	-1.6314			*L*_*OSD*_	-1.3657
		*L*_*OSD+*_	0.0367			*L*_*OSD+*_	0.0133
		*L*_*OSD*−_	0.2537			*L*_*OSD*−_	0.1956
		*W*_*LR*_	-1.5988			*W*_*LR*_	-1.3425
				*W*_*SR*_	2.7631***	*W*_*SR*_	3.3897***
ARCH	22.4499***	ARCH	19.1295**	ARCH	20.5782***	ARCH	23.0491***
*Adj*−*R*^2^	0.1141	*Adj*−*R*^2^	0.1200	*Adj*−*R*^2^	0.0580	*Adj*−*R*^2^	0.1266

***, **,and*denote significance at 1%, 5%, and 10% level, respectively.

## Discussion

Oil prices play a critical role in the global economy. Thus, it is important to understand the determinants and effects of oil prices. Although the relationship between oil price and investor sentiment has attracted more and more attentions recently, there has been no empirical research focusing on the links between the decomposed oil shocks (oil supply, aggregate demand shock, and oil-specific demand) and investor sentiment. This paper fills the gap.

The crude oil price shocks are firstly distinguished using the SVAR model, and then the linear and nonlinear causalities between the different oil shocks and investor sentiment are examined. We find that, there exists a significant linear nonlinear Granger cause from oil-specific demand shock to investor sentiment while causalities cannot be found among other variables, indicating only the changes of oil-specific demand shock can lead to the changes of investor sentiment. Moreover, there is no significant relationship between other oil shocks (including supply shock and aggregate demand shock) and investor sentiment. Based on these causality results, the time-varying and asymmetric effects of the oil-specific demand shock on investor sentiment are further examined by using the TVP-VAR model and the NARDL model, respectively. Furthermore, we demonstrate that the oil-specific demand shock has a persistent and time-varying positive effect on investor sentiment in general, except for the period during 1990–1992 when the oil-specific demand shock performs a small negative influence on investor sentiment due to the Gulf War. Finally, our findings suggest that oil-specific demand shock has a short-run rather than a long-run asymmetric effect on investor sentiment. Specifically, it is the negative oil-specific demand shock instead of the positive one has a significant positive effect on investor sentiment in the short-run.

Our findings have important implications. Firstly, this study contributes to the current theories on the interaction between oil shocks and investor sentiment. The oil-specific demand shock, as an important factor in investor sentiment, can facilitate investors’ decisions. Secondly, the price of oil and its shocks are key variables for policy makers, and understanding the degree to which shocks in oil prices affect investor sentiment will enable policymakers to generate more accurate forecasts to minimize potential business cycle fluctuations. An additional novelty of this work is that use the TVP-VAR and NARDL approach offers a comparative capacity to identify estimated links and could not be, otherwise, revealed through regressions, and the results have theoretical implications. On one hand, since the impact of oil-specific demand shock on investor sentiment is time-dependent, it is necessary for relevant departments and investors to adjust the strategies or decision making over time. On the other hand, as there are significant short-run asymmetric effects from oil-specific demand shock to investor sentiment, and their negative shocks have greater effects than positive shocks do. This tells government policy makers should response quickly by suing policy instruments to stabilize investor sentiment when the oil-specific demand shock shows high, especially negative fluctuations.

A potential extension of the current study is to investigate the relationship between oil price shocks and other investor sentiment measures in U.S market, such as American Association of Individual Investor (AAII). And it would be interesting to examine the impacts of oil price shocks on different types of sentiment, such as positive sentiment and negative sentiment. Furthermore, separating the US and non-US oil price shocks could be an interesting avenue for future research. Future research would also investigate as to the dynamic interaction between oil price shocks and investor sentiment in other countries, and examine whether there exist differences results in different countries. Finally, given the increased importance of investor sentiment in economic and financial decision making, it is important to examine the ability of oil price shock to improve the forecasting accuracy of investor sentiment and other related indicators.
